# Supplementary material for: Lactobacillus acidophilus (LA) Fermenting Astragalus Polysaccharides (APS) Improves Calcium Absorption and Osteoporosis by Altering Gut Microbiota
Source: Foods. 2023 Jan 6;12(2):275. doi: 10.3390/foods12020275 (PMC9858548; doi:10.3390/foods12020275)
Supplement: Supplementary file 1 [file foods-12-00275-s001.zip › foods-2123939-supplementary.pdf]

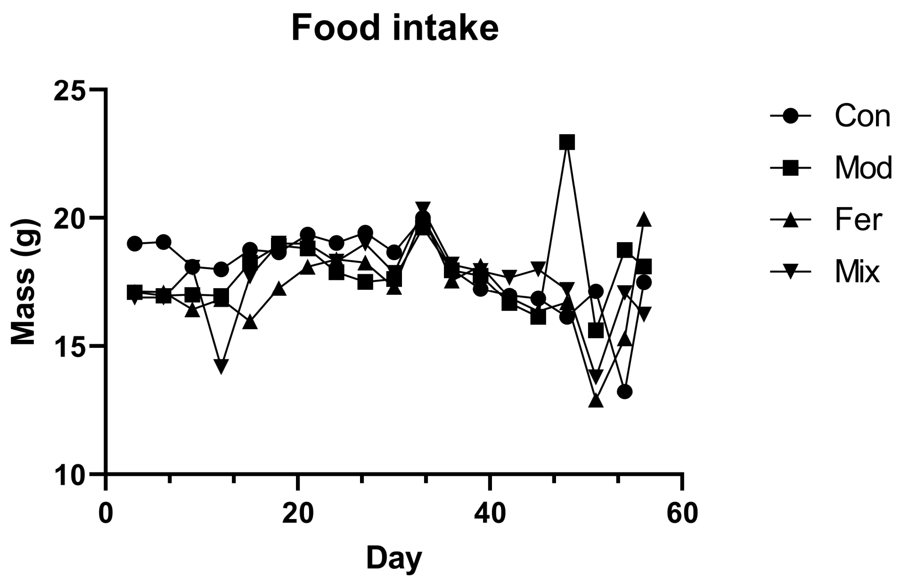

Supplementary Figure S1. Food intake of rats in each group.

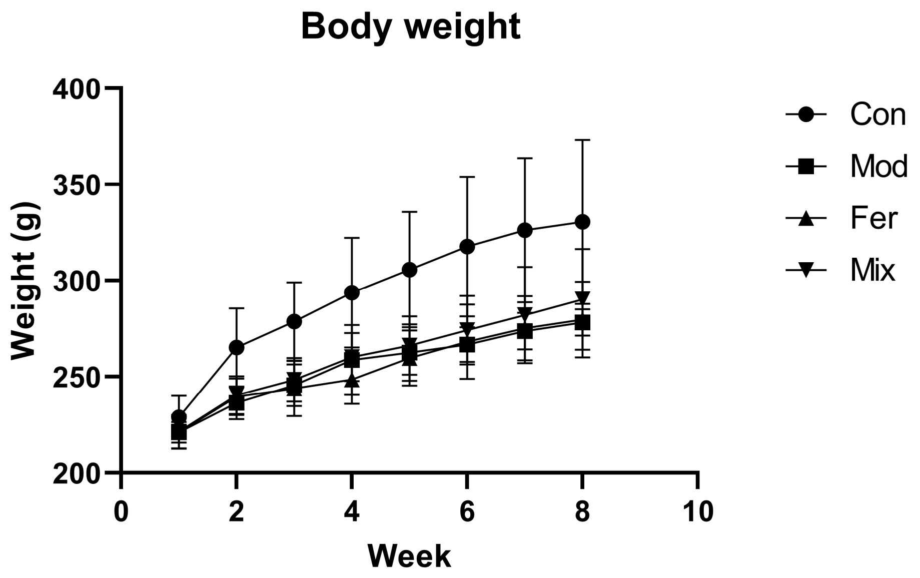

Supplementary Figure S2. Body weight of rats in each group.
